# Identification of Prognostic Biomarkers of Ovarian High-Grade Serous Carcinoma: A Preliminary Study Using Spatial Transcriptome Analysis and Multispectral Imaging

**DOI:** 10.3390/cells14100681

**Published:** 2025-05-08

**Authors:** Haeyoun Kang, Je-Gun Joung, Hyun Park, Min Chul Choi, Doohyun Koh, Ju-Yeon Jeong, Jimin Lee, Sook-Young Kim, Daun Jung, Sohyun Hwang, Hee Jung An

**Affiliations:** 1 Department of Pathology, CHA Bundang Medical Center, CHA University School of Medicine, Seongnam 13496, Republic of Korea; hykang@cha.ac.kr; 2 Department of Biomedical Science, CHA University, Seongnam 13496, Republic of Korea; jgjoung@cha.ac.kr (J.-G.J.); timoplay12@naver.com (D.K.); 3 CHA Future Medicine Research Institute, CHA Bundang Medical Center, CHA University School of Medicine, Seongnam 13496, Republic of Korea; dufakthd@chamc.co.kr (J.-Y.J.); c094002@chamc.co.kr (J.L.); skylove012@chamc.co.kr (S.-Y.K.); 4 Department of Biomedical Informatics, CHA University, Seongnam 13496, Republic of Korea; 5 Department of Gynecological Oncology, Comprehensive Gynecologic Cancer Center, CHA Bundang Medical Center, CHA University School of Medicine, Seongnam 13496, Republic of Korea; p06162006@cha.ac.kr; 6 Department of Obstetrics and Gynecology, Asan Medical Center, University of Ulsan College of Medicine, Seoul 05505, Republic of Korea; oursk@naver.com; 7 CHA Biomedical Research Institute, CHA Bundang Medical Center, Seongnam 13496, Republic of Korea; jhd2800@chamc.co.kr

**Keywords:** ovarian cancer, biomarkers, spatial transcriptomics, cancer recurrence, tumor microenvironment

## Abstract

Ovarian cancer is a lethal malignancy, with most patients initially responding to chemotherapy but frequently experiencing recurrence. Previous studies primarily examined tumor characteristics using limited genetic markers or bulk RNA sequencing. Here, we used spatial transcriptomics via the GeoMx^®^ platform, alongside multispectral immune cell immunofluorescence (IF), to identify biomarkers associated with disease progression following first-line treatment of high-grade serous carcinoma (HGSC). We identified several spatial biomarkers linked to non-recurrence, including elevated *NKG7* expression in CD45+ immune cell regions (*p* = 0.0011) and higher *TFPI2* and *PIGR* expression in tumor areas (*p* = 2.09 × 10^−6^), both associated with improved progression-free survival. Multispectral IF revealed significantly higher regulatory T cell (Treg) to CD8+ T cell ratios in the tumor nests and stroma of recurrent patients (*p* = 0.016, 0.048). Tregs were also found closer to cancer cells or macrophages than CD8+ T cells in recurrent tumors (*p* = 0.048), correlating with poor survival. Integrated analysis showed that immune cell density and immune pathway scores in the recurrent group positively correlated with cancer pathway scores, except for NF-κB. This comprehensive analysis revealed clues to interactions between different immune cells and identified biomarkers that may be useful for predicting recurrence of HGSC.

## 1. Introduction

Ovarian cancer (OC) is a highly lethal gynecological malignancy; indeed, it is one of the most common causes of cancer-related mortality in women worldwide [1]. Although most OC patients are responsive to chemotherapy, they frequently present with recurrent metastatic lesions that result in poor overall survival [2]. Therefore, precise diagnosis and treatment based on distinctive clinical, molecular, and immunologic characteristics of the tumor in each patient are imperative for strategic treatment planning and accurate prediction of recurrence.

High-grade serous carcinoma (HGSC), the most common and deadliest type of OC, is characterized by its unique clinical, genomic, and immunologic profile and high rates of recurrence. HGSCs present with a complex landscape of cellular and molecular diversity, with varied morphological patterns and tissue architectures within individual tumors [3]. The molecular profile of these cancers is characterized by a high frequency of TP53 tumor suppressor gene mutations coupled with chromosomal instability, resulting in extensive copy number variations [4,5]. The prognosis of HGSC is affected significantly by the immune profile of the tumor microenvironment (TME) [6]. These immune profiles within the TME are associated with survival outcomes and/or tumor recurrence. Crucially, the TME in HGSC patients displays remarkable variability, not only between patients but also among distinct tumor sites within a single patient, and even across different regions within an individual tumor. This huge diversity underscores the need for spatial transcriptomic approaches to comprehensively map and understand the intricate interactions between tumor cells and immune components across various tumor regions, thereby providing a more nuanced understanding of HGSC biology and potentially guiding more effective, personalized treatment strategies.

Recently, spatial transcriptomics approaches to assessing the localization of mRNA molecules within the tissue architecture of histological sections have been developed. These approaches provide valuable insight into the spatial characteristics of gene expression within tissues and are a promising method for discovering novel biomarkers for disease progression and treatment responses. Among the different types of spatial transcriptomics approaches, we utilized the GeoMx^®^ Digital Spatial Profiling (DSP) System, which can visualize formalin-fixed paraffin-embedded (FFPE) tissue sections stained with fluorescently labeled antibodies. Regions of interest (ROI) within the tissue are selected prior to spatial RNA profiling of multiple genes [3]. The capability for a simultaneous high-level multiplex approach to examining tissue areas of interest, selected on the basis of marker expression, makes DSP suitable for analyzing multiple biological markers within specific areas of a tissue sample at the same time.

The aim of this study was to use this spatial transcriptome analysis approach to identify biomarkers associated with the progression of HGSCs after first-line treatment. We investigated diverse gene signatures related to immune infiltration, taking into consideration their spatial enrichment patterns. Furthermore, we used multispectral imaging assessment (i.e., multiplex immunofluorescence (IF) analysis) by the Vectra Polaris™ system (Akoya Bioscience, Marlborough, MA, USA) to computationally identify and quantify different immune cell types within the tumor and stromal regions. This approach also allowed us to analyze the distance between cancer cells and different types of immune cells. By integrating these two methods, we sought to gain detailed insight into the spatial dynamics of gene expression, as well as immune cell interactions within the TME. A comprehensive understanding of these interactions is critical for improving treatment methods and enabling a personalized approach to this type of OC.

## 2. Materials and Methods

### 2.1. Patients and Construction of Tissue Microarray (TMA)

This study examined fresh and paraffin-embedded tissues from 12 cases selected from the archives; these patients had HGSC and were treated with standard therapy (radical hysterectomy with bilateral salpingo-oophorectomy, followed by platinum-based chemotherapy) at CHA Bundang Medical Center between 2007 and 2014. This study was approved by the institutional review board of CHA Bundang Medical Center (IRB #CHAMC 2020-03-004). Patient informed consent was received. The biospecimen and data used in this study were provided by Bundang CHA Biobank, Korea Biobank Network (KBN4-A05).

For NanoString GeoMx^®^ DSP (NanoString Technologies, Seattle, WA, USA), a TMA was constructed after two board-certified pathologists (HK and HA) reviewed representative whole slide tumor sections and provided a histologic diagnosis according to WHO classifications (2020) [7]. One representative core (5 mm diameter) was obtained from a representative FFPE block of tissue from each case. Table 1 summarizes the clinicopathologic characteristics of all 12 patients.

### 2.2. Spatial Transcriptome Profiling

The NanoString GeoMx^®^ DSP (NanoString Technologies, Seattle, WA, USA) was used to identify and visualize tumor cells, immune cells, and stromal cells. Each of these areas was viewed by immunofluorescent staining of morphology markers: PanCK for tumor tissue, CD45 for immune cells, and SMA for stroma. DAPI was used to visualize cell nuclei. In 11 cases, two rectangular-shaped ROIs per core were annotated by a board-certified pathologist (HK), whereas one ROI was annotated within a smaller piece of tumor tissue from one case (Patient 12). Each rectangular ROI had an area of 660 × 785 μm^2^. In total, 23 ROIs were annotated, each containing three areas of interest (AOIs) showing expression of the selected morphologic markers (i.e., PanCK, SMA, and CD45). In total, 69 AOIs were generated by automated tissue segmentation conducted using NanoString GeoMx^®^ DSP software and based on expression of the three morphology markers. The data were confirmed by a board-certified pathologist (HK).

After automated tissue segmentation, oligonucleotides were released from the selected ROIs upon localized exposure to UV light. Photocleaved oligonucleotides were collected via microcapillary aspiration and dispensed into a 96-well plate. Localized UV exposure and PC-oligonucleotide collection were conducted for each ROI. Oligonucleotides collected from each AOI were amplified by Polymerase Chain Reaction (PCR). The PCR products were pooled and purified using AMPure XP beads (Beckman Coulter Diagnostics Inc., Brea, CA, USA). Purified libraries were pooled and sequenced separately. Library quantity and quality were measured using a Qubit 4.0 (Thermo Fisher Scientific, Waltham, MA, USA) and TapeStation 4200 (Agilent Technologies, Santa Clara, CA, USA). Sequencing was performed on a NovaSeq 6000 platform, with 38 bp paired-end reads (Illumina, San Diego, CA, USA). The whole transcriptome within each AOI within a selected ROI was analyzed.

### 2.3. Spatial Transcriptome Data Processing

Sequencing for spatial transcriptome data was conducted using the GeoMx^®^ NGS Pipeline. After sequencing, reads were aligned to identify the unique ID of the probe. Duplicate reads were removed using the unique identifier region, and then the reads were converted to digital counts. The presence or absence of a gene was defined by the limit of quantitation (LOQ), which is the geometric mean of the negative probes multiplied by the geometric standard deviation raised to the second power. Of the 18,677 genes assayed, 16,132 (86.4%) were above the LOQ in 10% of the AOIs. Q3 normalization (75% of counts in each sample) was used for downstream analysis to account for variations in the area of the AOIs.

### 2.4. Gene Expression Analysis

The SpatialDecon R package v1.6.0, which performs deconvolution of mixed cells from spatial gene expression data based on constrained log-normal regression, was used to estimate cell abundance based on the spatial gene expression dataset derived from each AOI [8]. The SafeTME matrix, a cell profile matrix used to estimate immune and stromal cell types in tumors, was used for deconvolution [5]. DEGs were identified using the edgeR R package [6]. For multiple comparisons, we used the Benjamini–Hochberg *p*-value correction procedure. Gene Ontology analysis was performed using the clusterProfiler R package v4.4.4 [9]. Data were visualized as a pie chart constructed using the ggplot2 R package v3.5.1.

### 2.5. Multispectral Imaging of Immune Cells Using VECTRA

Multispectral staining was performed using a multiplex immunohistochemistry (IHC) method, followed by the generation of fluorescent images using the fluorophores contained in the Opal 7 kit (Akoya Biosciences, Marlborough, MA, USA). Multispectral staining was conducted using an automated staining apparatus (Bond Rx, Leica Biosystems, Nussloch, Germany) and specific antibodies (see below). After staining, whole slide scanning was performed to obtain images (magnification ×20) using Vectra Polaris™ (Akoya Biosciences, Marlborough, MA, USA). Target cells were identified based on protein expression levels, and autofluorescence was minimized using control tissues. A fluorescence cut-off level was established for certain target proteins, and the optimal cut-off value was derived using multiple analyses of tissues and receiver operating characteristics curve analysis. Quantification of the fluorescent images was performed on a pixel-by-pixel basis using Phenochart v1.1.0 within inForm^®^ software 2.5.1 (Akoya Bioscience, Marlborough, MA, USA). The resulting numeric data facilitated two-dimensional differentiation of tumor nests and non-neoplastic stroma within the TME. Classification of immune cells within the TME was performed using cell type-specific antibodies, including anti-CD8 (for cytotoxic T cells), anti-PD1 (an immune checkpoint inhibitor), anti-CD68 (for macrophages), anti-FOXP3 (for Treg cells), anti-CD56 (for NK/T cells), anti-CK (for carcinoma cells), and DAPI (for cell nuclei). Cell density and the distance between cell types in the tumor nests and stroma were then analyzed.

### 2.6. Integrative Analysis of Multispectral Immune Cell and Spatial Transcriptome Data

The score for each identified immune and cancer cell pathway was calculated by the GSVA R package v1.46.0 [10] using gene sets derived from the mSigDB hallmark gene sets database v.7.5.1 [11] and WikiPathways v7.5.1 [12]. Pathways related to NF-κB signaling, Warburg effect signaling, RAS signaling, Wnt signaling, and autophagy were identified from the WikiPathways database; all other pathways were identified from the mSigDB hallmark gene sets database. Correlations between gene expression, pathway scores, and immune cell density were calculated by Spearman’s correlation analysis. Correlation plots were constructed using corrplot within the R package.

### 2.7. Statistical Analysis

Survival analysis was performed using a Cox proportional hazards model within the survival R package. The statistical comparison of data from the two groups was performed using the Wilcoxon rank sum test. All plots were drawn in the ggplot2 package in R. *p* values < 0.05 were considered significant.

## 3. Results

### 3.1. Spatial Transcriptome Analysis and Gene Profiling of Selected ROI

To identify gene profiles associated with immunogenetic responses in HGSC patients, we used the NanoString GeoMx^®^ DSP to examine TMA slides from 12 patients (four non-recurring and eight recurring cases) who received standard treatment. The four patients without recurrence were named the “non-recur group” and the eight patients with recurrence were named the “recur group”. The tumor tissue, immune cells, and stromal regions were visualized using fluorescently labeled antibodies specific for PanCK (for the tumor area), CD45 (for the immune cell area), and SMA (for the stroma area). In total, 23 ROIs were selected for RNA sequencing (average 1.9 ROIs per patient).

**Figure 1:**
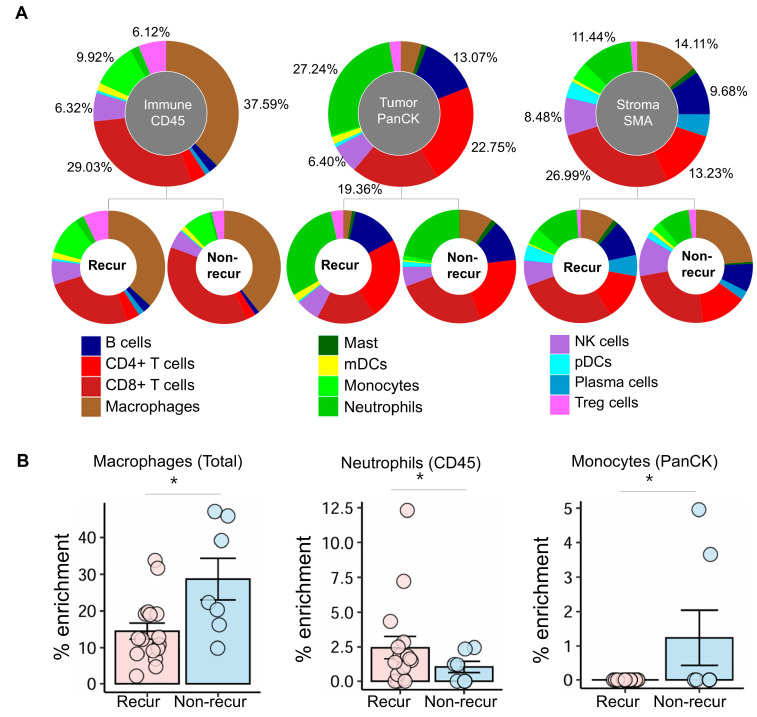
The proportions of cell types within each of the captured areas in ovarian cancer tissue sections. (**A**) The percentage of each cell type in the tumor (PanCK), immune (CD45), and stromal (SMA) areas within the tumor microenvironment, depicted as pie charts. The upper pie charts display combined data from all samples, whereas the bottom charts show data specific to each area in the recur and non-recur groups. (**B**) Comparison of macrophages in the total three areas, neutrophils in the CD45 (immune cell) area, and monocytes in the PanCK (tumor) area. mDCs: myeloid dendritic cells, pDCs: plasmacytoid dendritic cells and *: *p* < 0.05.

The deconvolution method was used to estimate the proportion (%) of each immune cell type in each area within the selected ROI for each HGSC sample (Figure 1A and Supplementary Table S1). Each area showed distinctive characteristics with respect to cell-type enrichment. In the CD45 immune cell area, we found enrichment of mainly CD8+ T cells and macrophages (29.03% and 37.59%, respectively). In the PanCK tumor area, we found that the percentages of CD4+ T cells, CD8+ T cells, and neutrophils tended to be higher than those of other cell types (22.75%, 19.36%, and 27.24%, respectively). In the stroma area, the percentages of CD8+ T, CD4+ T cells, and macrophages were highest (26.99%, 13.23%, and 14.11%, respectively). The percentage of macrophages across the entire ROI was higher in the non-recur group than in the recur group (28.70% vs. 14.48%, respectively; *p* = 0.015) (Figure 1B). When we compared differences in the percentages of the different cell types within each area between the non-recur and recur groups, we found that the percentage of monocytes in the tumor area was significantly lower in the recur group than in the non-recur group (0.00% vs. 1.23%, respectively; *p* = 0.034). Among the CD45 immune cell population, we found that the percentage of neutrophils was significantly higher in the recur group than in the non-recur group (2.58% vs. 0.71%, respectively; *p* = 0.03).

### 3.2. Identification of Differentially Expressed Genes (DEGs) Between the Non-Recur and Recur Groups

**Figure 2:**
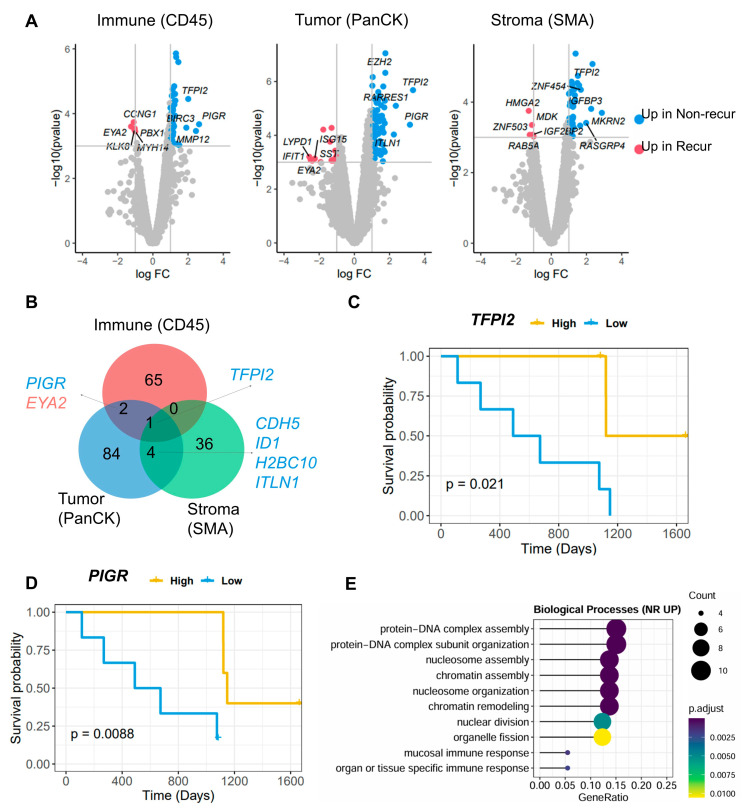
Differences in transcriptomic data between the recur and non-recur groups. (**A**) Volcano plots showing DEGs in each area, and (**B**) Venn diagram identifying overlapped DEGs. (**C**) Progression-free survival (PFS) analysis between high and low groups of *TFPI2* expression, and (**D**) *PIGR* expression. (**E**) Enriched Gene Ontology terms associated with up-regulated genes in the PanCK area of the non-recur (NR) than in that of recur groups. NR UP: Up-regulated in non-recur group and *p*. adjust: adjusted *p*-value.

DEGs (|log_2_FC (fold change) | ≥ 1.0 and *p* < 0.001) in each area within the recur and non-recur groups were identified (Figure 2A,B and Supplementary Table S2). Among the DEGs, *TFPI2* showed the greatest overexpression in the non-recur group (*p* = 2.09 × 10^−6^ in the tumor area); indeed, expression of this gene was highest across all three areas in this group. Higher expression of this gene was associated with a better prognosis in the non-recur group (progression-free survival (PFS), *p* = 0.021; median cut-off = 13.86 (immune area); Figure 2C). Expression of *PIGR* was significantly higher in the non-recur group, and was associated with a better prognosis (PFS, *p* = 8.8 × 10^−3^; median cut-off = 17.67 (immune areas), Figure 2D). By contrast, the expression of *EYA2* was higher in the immune cells and tumor areas of the recur group. *CDH5*, *ID1*, *H2BC10*, and *ITLN1* were common DEGs in the tumor and stroma areas. Next, we conducted Gene Ontology analysis to identify enriched functions in the tumor and stroma areas (adj. *p*-value < 0.05; Supplementary Tables S3 and S4). The data revealed that biological functions such as ‘protein-DNA complex assembly’, ‘chromatin remodeling’, and ‘organ- or tissue-specific immune response’ (adj. *p*-value = 1.69 × 10^−7^, 1.67 × 10^−5^, and 0.003, respectively) were enriched in the tumor area of the non-recur group (Figure 2E).

### 3.3. Identification of TME-Related Markers

**Figure 3:**
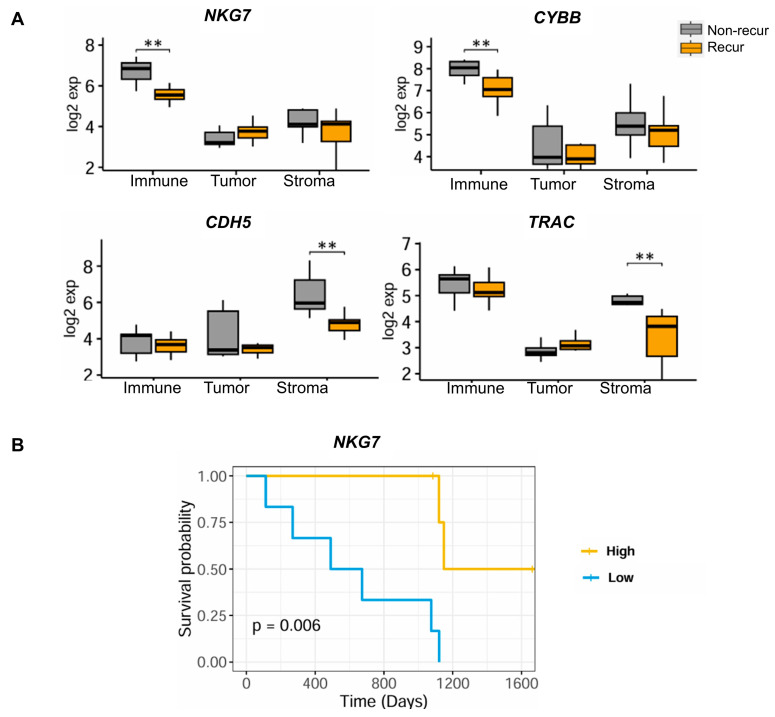
Expression and prognosis related to the expression of TME-associated genes. (**A**) Comparison of the expression of genes associated with TME in the recur and non-recur groups. (**B**) Analysis of PFS related to the expression of *NKG7*. The expression level is classified as low or high based on the median cut-off value. ** *p* < 0.01.

To identify potential therapeutic targets related to tumor recurrence and survival, we examined common genes between DEG genes and the pan-cancer microenvironment gene set suggested by Bagaev et al. [13]. For each immune cell, tumor, and stroma area, we examined statistically significant differences in gene expression between the non-recur and recur groups (Figure 3A). In the immune area, *NKG7* and *CYBB* were significantly overexpressed in the non-recur group (Log_2_FC = 1.32 and 1.12 at *p* = 1.79 × 10^−6^ and 1.77 × 10^−5^, respectively). Two genes (*CDH5* and *TRAC*) showed higher expression in the stroma area of the non-recur group (Log_2_FC = 1.28 and 1.23 at *p* = 4.23 × 10^−6^ and 2.60 × 10^−4^, respectively). Analysis of PFS related to the genes showing significantly higher or lower expression in the recur and non-recur groups (Supplementary Figure S1) revealed that high expression of *NKG7* in the immune area was associated with a favorable prognosis (*p* = 6.0 × 10^−3^, median cut-off = 53.5) (Figure 3B).

### 3.4. Multispectral Quantitative Computational Assessment of Immune Cell Density and Distance Between Tumor and Immune Cells Within Tumor Nests and Stroma

To examine and visualize the distribution of immune cells within HGSC tissue, we used multiplex IF markers to identify these cells by detecting expression of the immune checkpoint molecule PD1 expressed by CD8+ T cells as well as other immune cells. The density and spatial distribution (i.e., distance) between CD8+ T cells, which are pivotal for tumor cell cytotoxicity, and CD68+ macrophages, particularly tumor-associated macrophages that play a critical role in the TME, were analyzed. Our earlier analyses of cell percentages in the different areas identified both of these cell types as predominant components. In addition to PD1-CD8+ T cells and PD1+CD8+ T cells, we analyzed regulatory T cells (FOXP3+), which contribute to the suppressive TME through their actions on CD8+ T cells, macrophages, and cancer cells.

**Figure 4:**
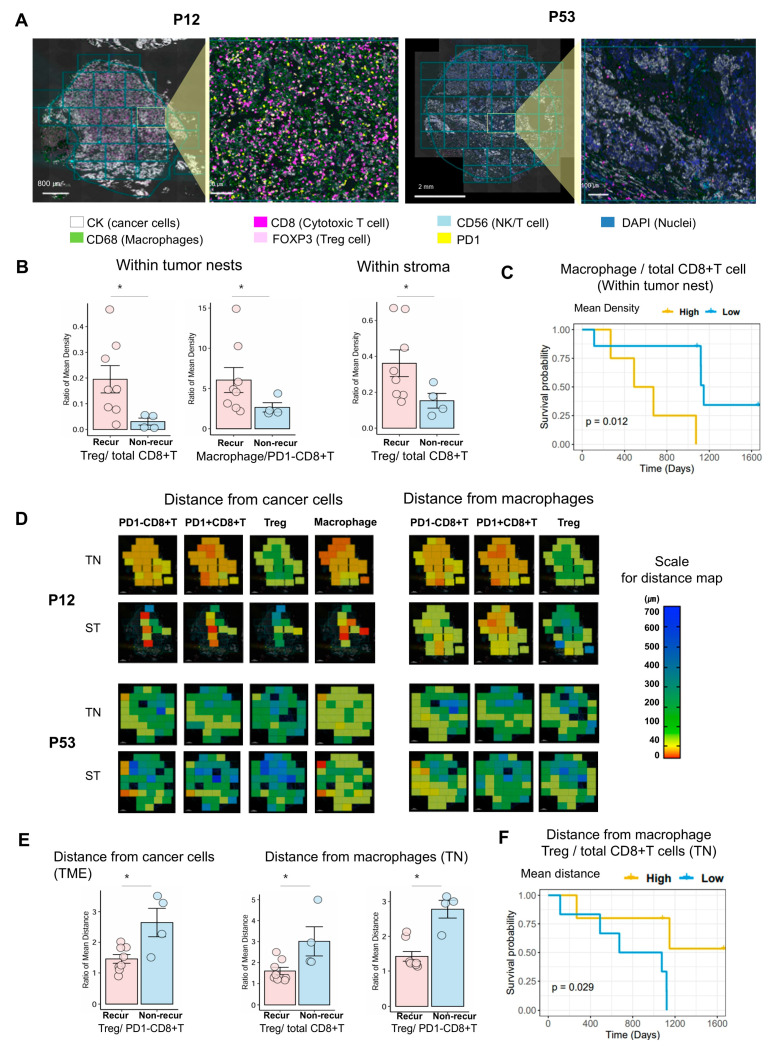
Comparison of mIF immune cell density, and distances between cell types in the non-recur and recur groups. (**A**) mIF immune cell densities of representative patients of recur (P53) and non-recur (P12) groups. Many immune cells, including CD8+ T cells and macrophages, were highly infiltrated in P12, whereas they were scant in P53. (**B**) Comparison of mIF immune cell density between the two groups. The box plots show the Treg/PD1-CD8+ T cell ratio and the macrophage/PD1-CD8+ T cell ratio in tumor nests, and the Treg/total CD8+ T cell ratio in the stroma. (**C**) PFS difference between high and low groups of macrophage/total CD8+ T cell in mIF immune cell density. (**D**) Representative images showing the mean distance from cancer cells to the nearest immune cell. The distances between cancer cells and immune cells, and the distance between macrophages and immune cells in P12 (non-recur group) were much shorter (red color) than in P53 (recur group). (**E**) Comparison of the mIF immune cell mean distance data from the two groups. Data depict the Treg/PD1-CD8+ T ratio in the TME, and Treg/total CD8+ T cell and Treg/PD1-CD8+ T cell ratios in tumor nests. (**F**) PFS difference between high and low groups of Treg/total CD8+ T cell (tumor nest, TN) in mIF immune cell mean distance from macrophages. * *p* < 0.05.

The overall mean density of total CD8+ T cells, PD1-CD8+ T cells, PD1+CD8+ T cells, regulatory Treg cells, and macrophages was higher in both tumor nests and stromal areas in the non-recur group than in those of the recur group (Supplementary Table S5). The mean CD8+ T cell density in the tumor nest and stromal areas of the non-recur group was higher than in that of the recur group (289.0 vs. 46.6 and 320.7 vs. 110.0, respectively); however, the difference was not statistically significant. The mean macrophage density in the tumor nest and stromal area of the non-recur group was higher than in the recur group (270.3 vs. 137.2 and 443.2 vs. 332.5, respectively), but again, the difference was not statistically significant. Figure 4A shows a representative multiplex IF image of immune cells from a patient in the non-recur group (P12) and from a patient in the recur group (P53); the image shows high infiltration of CD8+ T cells and macrophages in the former, and scant infiltration by these cells in the latter. Although the difference in the overall mean density of immune cells between the recur and non-recur group was not statistically significant, the Treg/total CD8+ T cell ratio in both the tumor nest and stroma area was significantly higher in the recur group than in the non-recur group, by 6.40-fold (*p* = 0.016) and 2.37-fold (*p* = 0.048), respectively (Figure 4B and Supplementary Table S5). The Treg/PD1-CD8+ T cell and macrophage/PD1-CD8+ T cell ratios were also significantly higher (by 4.1-fold and 2.28-fold, respectively) in the tumor nest of the recur group than in that of the non-recur group (both *p* = 0.048, Supplementary Table S5).

When investigating the correlation between immune cell density and recurrence status, we found that the densities of most immune cells correlated positively with each other; however, the macrophage density in the stroma of the recur group correlated negatively with the density of other immune cells, especially CD8+ T cells and Treg cells (Supplementary Figure S2). Survival analysis in the context of immune cell density revealed that the group with a higher macrophage/total CD8+ T cell density ratio within the tumor cell nest, meaning that the macrophage density was higher than that of total CD8+ T cells, exhibited a significantly poorer prognosis (*p* = 0.012, mean cut-off = 4.35; Figure 4C).

We assumed that actively interacting cells ought to be closer together; therefore, we measured the shortest distance between immune cells and cancer cells. Representative images showing the distance from a cancer cell to the nearest immune cell in the non-recur and recur groups are presented (P12, non-recur; P53, recur) in Figure 4D. We found that the distance between cancer cells and immune cells in the non-recur group was shorter than in the recur group, although the difference was not statistically significant (i.e., recur/non-recur in total CD8+ T cells: 1.40-fold) (Supplementary Table S6). The Treg/PD1-CD8+ T cell distance ratio from cancer cells was significantly smaller for the recur group than for the non-recur group, by 0.55-fold (*p* = 0.048) (Figure 4E and Supplementary Table S6). In addition, we observed that the Treg/total CD8+ T cell distance from macrophages in the tumor nests was significantly shorter in the recur group than in the non-recur group, by 0.53-fold (*p* = 0.048) (Figure 4E and Supplementary Table S7). Survival analysis in the context of distance ratios revealed that a lower Treg/total CD8+ T cell distance within the tumor cell nest, which suggests that Treg cells are closer to macrophages than CD8+ T cells, was associated with a poorer prognosis (*p* = 0.029, mean cut-off = 1.80; Figure 4F).

### 3.5. Integrative Analysis of Multispectral Immune Cell Density and Spatial Transcriptome Data Related to the TME

Finally, we performed an integrative analysis of the spatial transcriptome data and multispectral quantitative immune cell assessment data. Before integrating the two data sets, we first checked whether data derived from GeoMx and multispectral quantitative analysis of immune cells were consistent; for example, CD68 expression by macrophages, and CD8A expression by total CD8+ T cells. Data from multispectral quantitative analysis revealed that the macrophage density in the tumor nest correlated strongly with CD68 expression in the tumor area, as assessed by GeoMx (correlation coefficient = 0.66, *p* = 0.02), and that total CD8+ T cell density in the tumor nest correlated positively with CD8A expression in the tumor area; however, the correlation was not statistically significant (correlation coefficient = 0.43, *p* = 0.17; Supplementary Figure S3). Therefore, we integrated these two data sets.

**Figure 5:**
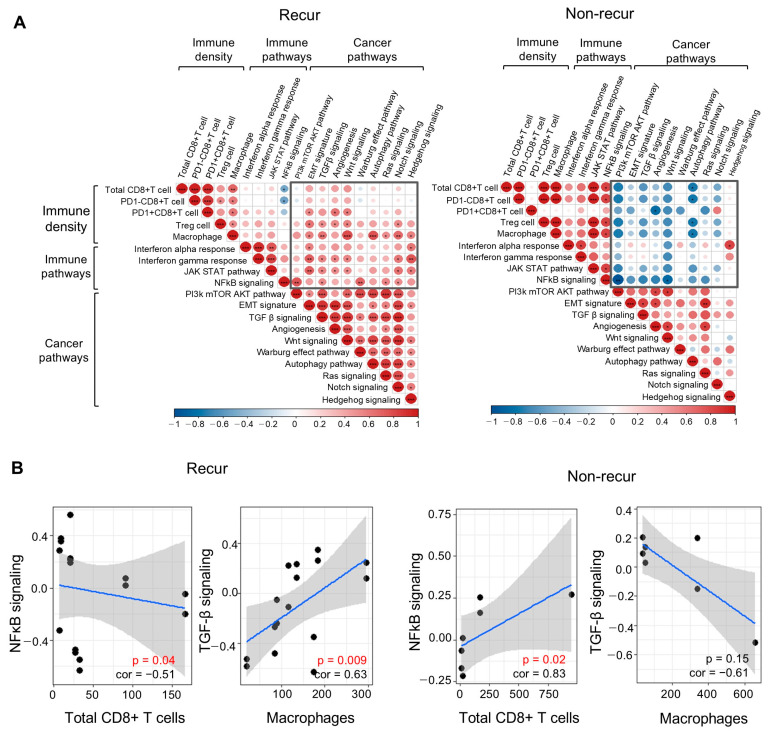
Correlation between the multispectral immune cell density and the immune and cancer pathway scores derived from the transcriptome data, based on recurrence status. (**A**) Correlation between immune cell density and the immune and cancer pathway scores derived from transcriptome data. The most significant differences are shown in black boxes. Colors represent the range of the correlation coefficient, from 1 (red) to −1 (blue). Statistical significance is denoted by an asterisk (* *p* < 0.05, ** *p* < 0.01, and *** *p* < 0.001). (**B**) Dot plots showing representative pathways (NF-κB signaling and TGF-β signaling pathway) and immune cell density (total CD8+ T cells and macrophages). The blue line and gray area represent the trend and the confidence interval of the correlation, respectively.

Upon investigating the correlation between immune cell density and the immune and cancer pathway scores derived from transcriptome data, we found that both immune cell density and immune pathway scores correlated positively with cancer pathway scores in the recur group; an exception was the NF-kB signaling pathway. However, in the non-recur group, immune cell density correlated positively with the immune pathway score, and negatively with most cancer pathway scores (including the PI3K/mTOR and Wnt signaling pathways; Figure 5). In addition, the NF-κB pathway score correlated negatively with total CD8+ T cells in the recur group, but positively in the non-recur group.

## 4. Discussion

The TME plays a crucial role in tumorigenesis; therefore, studying the TME in detail may provide comprehensive knowledge regarding responses to treatment [14]. In this study, we characterized gene expression profiles in the TME and examined their correlation with the recurrence of HGSC. We achieved this by conducting spatial transcriptome analysis of various histologic regions within FFPE slides of HGSC tissue. While bulk RNA sequencing has limitations with respect to representing the spatial diversity of tissue in cancer samples [15], spatial transcriptomics analysis of the tumor and TME provides more precise information that may give clues to the mechanisms underlying the recurrence of HGSC.

We identified several biomarkers that are associated with recurrence status. We found that overexpression of *TFPI2* and *PIGR* was associated with non-recurrence and a better prognosis. *TFPI2* (Tissue Factor Pathway inhibitor 2) encodes a member of the serine proteinase inhibitor family, which plays a multifaceted role in regulating protease activity, tissue remodeling, and cancer progression. *TFPI2* has been identified as a tumor suppressor gene in several types of cancer [16], and is a diagnostic marker for clear cell type OC; however, our results suggest that it could be a prognostic marker for HGSC. PIGR (Polymeric Immunoglobulin Receptor) is a transmembrane protein that transports polymeric IgA across the intestinal epithelium. A recent report shows that IgA-PIGR colonization is associated with improved survival of patients with HGSC [17], and IgA transcytosis through malignant epithelial cells via the IgA-PIGR interaction elicits transcriptional changes that sensitize tumor cells to cytolytic killing by T cells; therefore, PIGR might antagonize the growth of HGSC cells by regulating coordinated tumor cell, T cell, and B cell responses. *EYA2* (Eyes Absent Homolog 2) has been shown to be overexpressed in ovarian cancer cases and is associated with poorer survival outcomes, particularly in advanced-stage disease. Functionally, EYA2 acts as a transcriptional coactivator and has been shown to promote tumor growth in xenograft models [18].

In addition, among TME-related genes, we found that overexpression of *NKG7* in the immune cell area was associated with a favorable prognosis. NKG7 (natural killer cell granule protein-7) is associated with the quantity and trafficking (as well as calcium release) of cytolytic granules within cytotoxic CD8+ T cells, which are essential for effective anti-tumor cytotoxicity. NKG7 is regulated by the PD-1-NFAT signaling pathway; indeed, a previous report shows that patients with increased expression of NKG7 exhibit a complete response to anti-PD-1 therapy [19]. According to our results, increased expression of *NKG7* might also be associated with responses to conventional chemotherapy, as well as to PD1/PD-L1 immune checkpoint inhibitor therapy. In addition, we found that high expression of *NKG7* by immune cells correlated significantly with PFS of patients with HGSC, which, to the best of our knowledge, is the first report of a major role for NKG7 in HGSC.

We also performed multispectral quantitative IF assessments to examine the proximity between immune cells and cancer cells, as well as the density of each type of immune cell within the tumor and TME; this is because cells exhibiting closer proximity might be engaging in more active interactions. Our quantitative assessment revealed that the Treg/total CD8+ T cell ratio was significantly higher in the recur group than in the non-recur group. Treg cells suppress immune responses [20], and their presence often correlates with a poor prognosis because Tregs can promote tumor growth by suppressing the anti-tumor activity of CD8+ T cells [21]. Therefore, the ratio of Tregs to CD8+ T cells, rather than the density of Treg cells alone, could be a predictive marker for treatment responses in HGSC, a finding that agrees with previous reports [22,23,24]. Regarding the proximity of cells, we observed that the Treg/PD1-CD8+ T cell distance ratio from cancer cells, and that of Treg/total CD8+ T cells from cancer cells or macrophages, was significantly lower in the recur group than in the non-recur group (0.51-fold vs. 0.53-fold, respectively), which means that Treg cells are closer to cancer cells or macrophages than CD8+ T cells, and suggests that Treg cells in the recur group are more effective at suppressing the immune response than PD1-CD8+ or total CD8+ cytotoxic T cells are at killing cancer cells.

Next, we integrated the spatial transcriptomics data with the multispectral IF assessment data. Because multispectral immune cell densities were assessed in whole tissues, while spatial transcriptomics was conducted for only 69 regions within the same TMA, it was necessary to determine whether the assessment of these two different experimental datasets would introduce errors into the results of the integral analysis. To address this, we evaluated the correlation between the multispectral immune cell density and expression of the corresponding immune cell marker genes (e.g., CD68 for macrophages and CD8A for total CD8+ T cells) across the whole tissue area and in the overlapping regions. The results for the whole tissue area were highly consistent with those for the overlapping regions (Supplementary Figures S3 and S4), thereby confirming that our 69 selected regions were representative of the whole tumor tissue.

According to our integrated analysis of these spatial assessments, the immune cell density and immune pathways within the tumor nests of the recur group correlated positively with cancer pathways (with the exception of the NF-κB pathway). This finding implies that tumors with a TME in which the JAK/STAT or TGF-β signaling pathways in immune cells engage in crosstalk and positive interactions with other cancer signaling pathways, such as the PI3K/mTOR or Wnt signaling pathways, are prone to recurrence. By contrast, tumors exhibiting an inverse correlation between immune pathways and cancer pathways are less likely to recur. In addition, the NF-κB pathway correlated negatively with the total CD8+ T cell count in the recur group, but positively in the non-recur group. In general, NF-κB signaling plays a role in activating CD8+ T cells [25]; however, in some specific contexts and cellular environments, it may negatively impact CD8+ T cells. Possible explanations are that in some contexts, including cancer, activation of NF-κB is compromised by failure of the Rela (also known as p65, encoded by *Rela*) subunit in CD8+ T cells [26], or that CD8+ T cell responses can be modulated indirectly by Treg cells, which are also activated by NF-κB signaling [27]. However, we found that the TGF-β signaling pathway correlated positively with macrophages in the recur group, but negatively in the non-recur group, which might be related to the function of the TGF-β signaling pathway in promoting polarization of macrophages to the M2 phenotype, thereby creating an immunosuppressive environment and enhancing tumor progression.

Although this study is limited by the small number of cases, our novel integrated spatial analyses have profound implications not only for our understanding of the molecular tumor immune microenvironment in HGSC but also for the development of novel therapeutic targets for precision medicine approaches. In future studies, we will first investigate the potential of *NKG7* expression as a biomarker for immunotherapy responsiveness and examine the existence of tumor subclones associated with immune evasion. Finally, validation of these findings in a larger cohort is planned to strengthen the robustness and broader applicability of the results.

## 5. Conclusions

We identified the expression of *NKG7* by immune cells, as well as *TFPI2* and *PIGR* in the tumor area, as potential biomarkers that predict recurrence and survival of patients with HGSC. The Treg/total CD8+ T cell ratio and the distance ratio of Treg/total CD8+ T cells from cancer cells or macrophages are the other markers that predict recurrence and poorer survival. Interestingly, the correlation between immune cells and cancer pathways suggests that an immunosuppressive TME may be responsible for recurrence.

## Figures and Tables

**Table 1:** Clinicopathologic characteristics of 12 patients.

Patient ID	Age Group at Dx	Primary Site	Stage	Tumor Size	PFS > 5y	Recurrence Status	Lymph Node Metastasis	Distant Metastasis
Pat05	70s	Ovary	3c	14	Yes	Non-recurred	Absent	Absent
Pat09	40s	Ovary	3c	5.3	No	Recurred	Absent	Absent
Pat10	50s	Ovary	3c	7	No	Recurred	Present	Absent
Pat11	60s	Ovary	3b	6	Yes	Non-recurred	Absent	Absent
Pat12	50s	Ovary	2c	7.5	Yes	Non-recurred	Absent	Absent
Pat13	30s	Ovary	3c	4	Yes	Non-recurred	Present	Absent
Pat15	50s	Ovary	3c	10	No	Recurred	Present	Absent
Pat16	50s	Ovary	3c	12	No	Recurred	Present	Absent
Pat20	50s	Ovary	3c	10	No	Recurred	Present	Absent
Pat21	50s	Ovary	3c	12	No	Recurred	Present	Absent
Pat42	50s	Ovary	3c	15	No	Recurred	Present	Absent
Pat53	60s	Ovary	3c	12	No	Recurred	Present	Absent

Dx: Diagnosis.

## Data Availability

All expression data are available at the GEO Database (https://www.ncbi.nlm.nih.gov/geo/) (accessed on 3 May 2025) with accession number GSE279969.

## Supplementary Materials

The following supporting information can be downloaded at: https://www.mdpi.com/article/10.3390/cells14100681/s1, Figure S1: Progression-free survival based on expression of genes in the tumor microenvironment, Figure S2: Correlation between multispectral immune cell density and recurrence of HGSCs, Figure S3: Expression of immune cell marker genes in GeoMx spatial transcriptome data is similar to the corresponding mIF immune cell densities, Figure S4: Comparison of immune cell densities between overlapping ROIs and the whole tissue in the tumor nest with ex7pression of immune cell marker genes in GeoMx spatial transcriptome data; Table S1: The proportion (%) of cells in the immune (CD45), tumor (PanCK), and stromal (SMA) areas, Table S2: Differentially expressed genes in each compartment, Table S3: Enriched Gene Ontology terms for up-regulated genes in the tumor (PanCK) area, Table S4: Enriched Gene Ontology terms for up-regulated genes in the stroma (SMA) area, Table S5: Mean immune cell density according to recurrence status (/mm2), Table S6: Mean distance of cancer cells to the nearest immune cells (µm), Table S7: Mean distance of macrophages to the nearest immune cell (µm).

## Abbreviations

The following abbreviations are used in this manuscript.

adj.	adjusted
AOI	areas of interest
CDH5	cadherin 5
CYBB	cytochrome b-245 beta chain
DEGs	differentially expressed genes
DSP	Digital Spatial Profiling
Dx	diagnosis
FC	fold change
FFPE	formalin-fixed paraffin-embedded
HGSC	high-grade serous carcinomas
IF	immunofluorescence
LOQ	limit of quantitation
NKG7	natural killer cell granule protein 7
OC	ovarian cancer
PCR	Polymerase Chain Reaction
PFS	progression-free survival
PIGR	polymeric immunoglobulin receptor
ROI	regions of interest
TFPI2	tissue factor pathway inhibitor 2
TMA	tissue microarray
TME	tumor microenvironment
TN	tumor nest
TRAC	T cell receptor alpha constant
Tregs	regulatory T cells

## References

[1] Bray F., Laversanne M., Sung H., Ferlay J., Siegel R.L., Soerjomataram I., Jemal A. (2024). Global cancer statistics 2022: GLOBOCAN estimates of incidence and mortality worldwide for 36 cancers in 185 countries. CA Cancer J. Clin..

[2] Pogge von Strandmann E., Reinartz S., Wager U., Muller R. (2017). Tumor-Host Cell Interactions in Ovarian Cancer: Pathways to Therapy Failure. Trends Cancer.

[3] Zollinger D.R., Lingle S.E., Sorg K., Beechem J.M., Merritt C.R. (2020). GeoMx RNA Assay: High Multiplex, Digital, Spatial Analysis of RNA in FFPE Tissue. Methods Mol. Biol..

[4] Hohn A.K., Brambs C.E., Hiller G.G.R., May D., Schmoeckel E., Horn L.C. (2021). 2020 WHO Classification of Female Genital Tumors. Geburtshilfe Frauenheilkd.

[5] Danaher P., Kim Y., Nelson B., Griswold M., Yang Z., Piazza E., Beechem J.M. (2022). Advances in mixed cell deconvolution enable quantification of cell types in spatial transcriptomic data. Nat. Commun..

[6] Robinson M.D., McCarthy D.J., Smyth G.K. (2010). edgeR: A Bioconductor package for differential expression analysis of digital gene expression data. Bioinformatics.

[7] Herrington C.S. (2020). WHO Classification of Tumours Female Genital Tumours.

[8] Griswold M., Danaher P. (2022). SpatialDecon: Deconvolution of Mixed Cells from Spatial and/or Bulk Gene Expression Data. R package.

[9] Yu G., Wang L.G., Han Y., He Q.Y. (2012). clusterProfiler: An R package for comparing biological themes among gene clusters. OMICS.

[10] Hanzelmann S., Castelo R., Guinney J. (2013). GSVA: Gene set variation analysis for microarray and RNA-seq data. BMC Bioinform..

[11] Liberzon A., Birger C., Thorvaldsdottir H., Ghandi M., Mesirov J.P., Tamayo P. (2015). The Molecular Signatures Database (MSigDB) hallmark gene set collection. Cell Syst..

[12] Agrawal A., Balci H., Hanspers K., Coort S.L., Martens M., Slenter D.N., Ehrhart F., Digles D., Waagmeester A., Wassink I. (2024). WikiPathways 2024: Next generation pathway database. Nucleic Acids Res..

[13] Bagaev A., Kotlov N., Nomie K., Svekolkin V., Gafurov A., Isaeva O., Osokin N., Kozlov I., Frenkel F., Gancharova O. (2021). Conserved pan-cancer microenvironment subtypes predict response to immunotherapy. Cancer Cell.

[14] Yang Y., Yang J., Zhao X., Wei X. (2020). Tumor Microenvironment in Ovarian Cancer: Function and Therapeutic Strategy. Front. Cell Dev. Biol..

[15] Stur E., Corvigno S., Xu M., Chen K., Tan Y., Lee S., Liu J., Ricco E., Kraushaar D., Castro P. (2022). Spatially resolved transcriptomics of high-grade serous ovarian carcinoma. iScience.

[16] Kobayashi H., Imanaka S., Matsubara S., Shigetomi H., Yoshimoto C. (2024). Dual Role of Tissue Factor Pathway Inhibitor 2—A Novel Serodiagnostic Marker for Ovarian Cancer—In Human Cancers. Int. J. Transl. Med..

[17] Berntsson J., Lundgren S., Nodin B., Uhlen M., Gaber A., Jirstrom K. (2014). Expression and prognostic significance of the polymeric immunoglobulin receptor in epithelial ovarian cancer. J. Ovarian Res..

[18] Zhang L., Yang N., Huang J., Buckanovich R.J., Liang S., Barchetti A., Vezzani C., O’Brien-Jenkins A., Wang J., Ward M.R. (2005). Transcriptional coactivator Drosophila eyes absent homologue 2 is up-regulated in epithelial ovarian cancer and promotes tumor growth. Cancer Res..

[19] Wen T., Barham W., Li Y., Zhang H., Gicobi J.K., Hirdler J.B., Liu X., Ham H., Peterson Martinez K.E., Lucien F. (2022). NKG7 Is a T-cell-Intrinsic Therapeutic Target for Improving Antitumor Cytotoxicity and Cancer Immunotherapy. Cancer Immunol. Res..

[20] Noyes D., Bag A., Oseni S., Semidey-Hurtado J., Cen L., Sarnaik A.A., Sondak V.K., Adeegbe D. (2022). Tumor-associated Tregs obstruct antitumor immunity by promoting T cell dysfunction and restricting clonal diversity in tumor-infiltrating CD8+ T cells. J. Immunother. Cancer.

[21] Li C., Jiang P., Wei S., Xu X., Wang J. (2020). Regulatory T cells in tumor microenvironment: New mechanisms, potential therapeutic strategies and future prospects. Mol. Cancer.

[22] Baras A.S., Drake C., Liu J.J., Gandhi N., Kates M., Hoque M.O., Meeker A., Hahn N., Taube J.M., Schoenberg M.P. (2016). The ratio of CD8 to Treg tumor-infiltrating lymphocytes is associated with response to cisplatin-based neoadjuvant chemotherapy in patients with muscle invasive urothelial carcinoma of the bladder. Oncoimmunology.

[23] Hayashi Y., Ueyama A., Funaki S., Jinushi K., Higuchi N., Morihara H., Hirata M., Nagira Y., Saito T., Kawashima A. (2024). In situ analysis of CCR8^+^ regulatory T cells in lung cancer: Suppression of GzmB^+^ CD8^+^ T cells and prognostic marker implications. BMC Cancer.

[24] Preston C.C., Maurer M.J., Oberg A.L., Visscher D.W., Kalli K.R., Hartmann L.C., Goode E.L., Knutson K.L. (2013). The ratios of CD8+ T cells to CD4+CD25+ FOXP3+ and FOXP3- T cells correlate with poor clinical outcome in human serous ovarian cancer. PLoS ONE.

[25] Yatim N., Jusforgues-Saklani H., Orozco S., Schulz O., Barreira da Silva R., Reis e Sousa C., Green D.R., Oberst A., Albert M.L. (2015). RIPK1 and NF-kappaB signaling in dying cells determines cross-priming of CD8^+^ T cells. Science.

[26] Ling W., Rayman P., Uzzo R., Clark P., Kim H.J., Tubbs R., Novick A., Bukowski R., Hamilton T., Finke J. (1998). Impaired activation of NFkappaB in T cells from a subset of renal cell carcinoma patients is mediated by inhibition of phosphorylation and degradation of the inhibitor, IkappaBalpha. Blood.

[27] Hovelmeyer N., Schmidt-Supprian M., Ohnmacht C. (2022). NF-kappaB in control of regulatory T cell development, identity, and function. J. Mol. Med..

