# Supplementary material for: Identification of Prognostic Biomarkers of Ovarian High-Grade Serous Carcinoma: A Preliminary Study Using Spatial Transcriptome Analysis and Multispectral Imaging
Source: Cells. 2025 May 8;14(10):681. doi: 10.3390/cells14100681 (PMC12110039; doi:10.3390/cells14100681)
Supplement: Supplementary file 1 [file cells-14-00681-s001.zip › HGSC_Geomx_SFigure.pdf]

Supporting Information

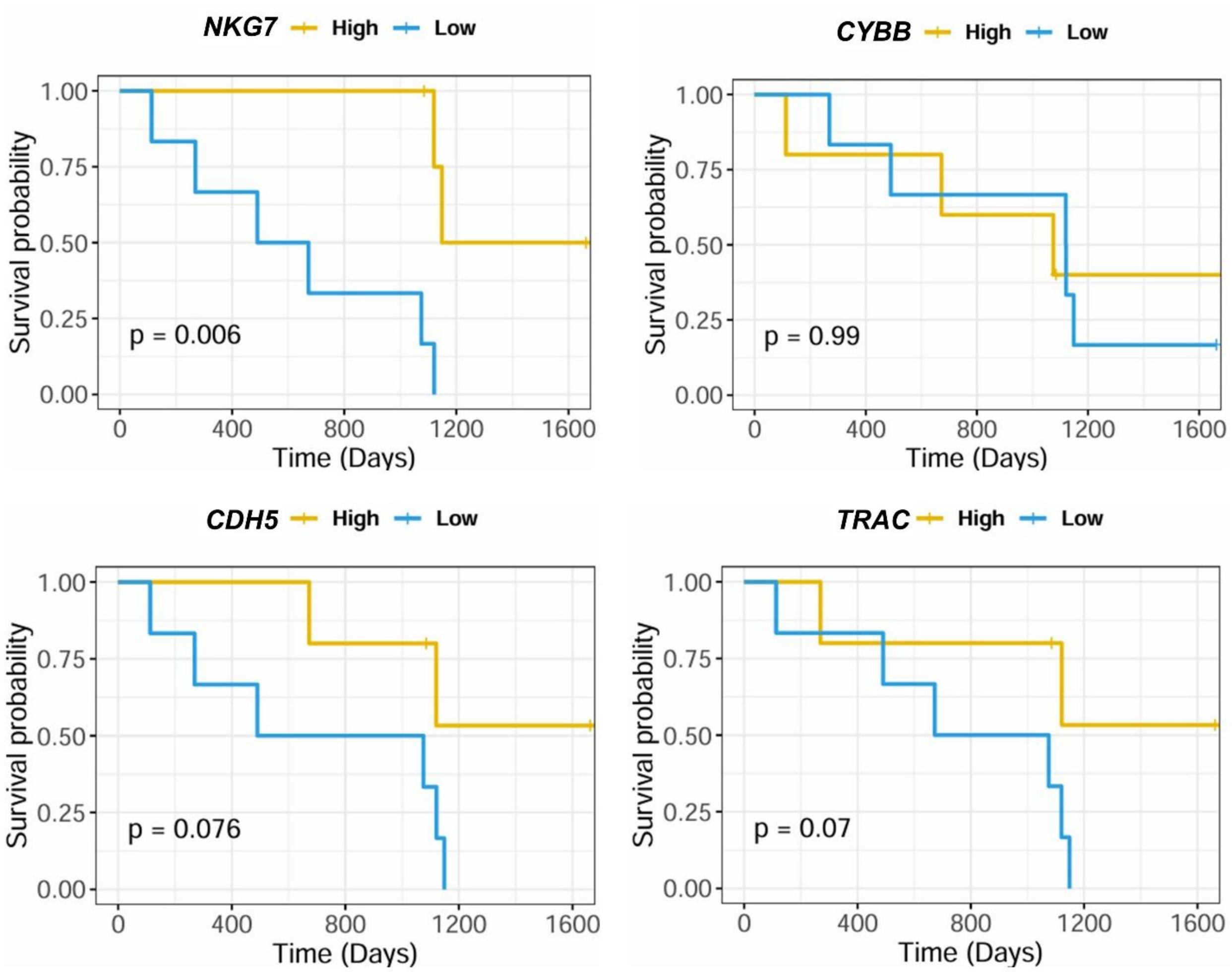

Supplementary Figure 1. Progression-free survival based on expression of genes in the tumor microenvironment. Survival of patients in the “high” and “low” expression groups, based on the median cut-off for *NKG7* and *CYBB* in the immune area, and *TRAC* and *CDH5* in the stroma area.

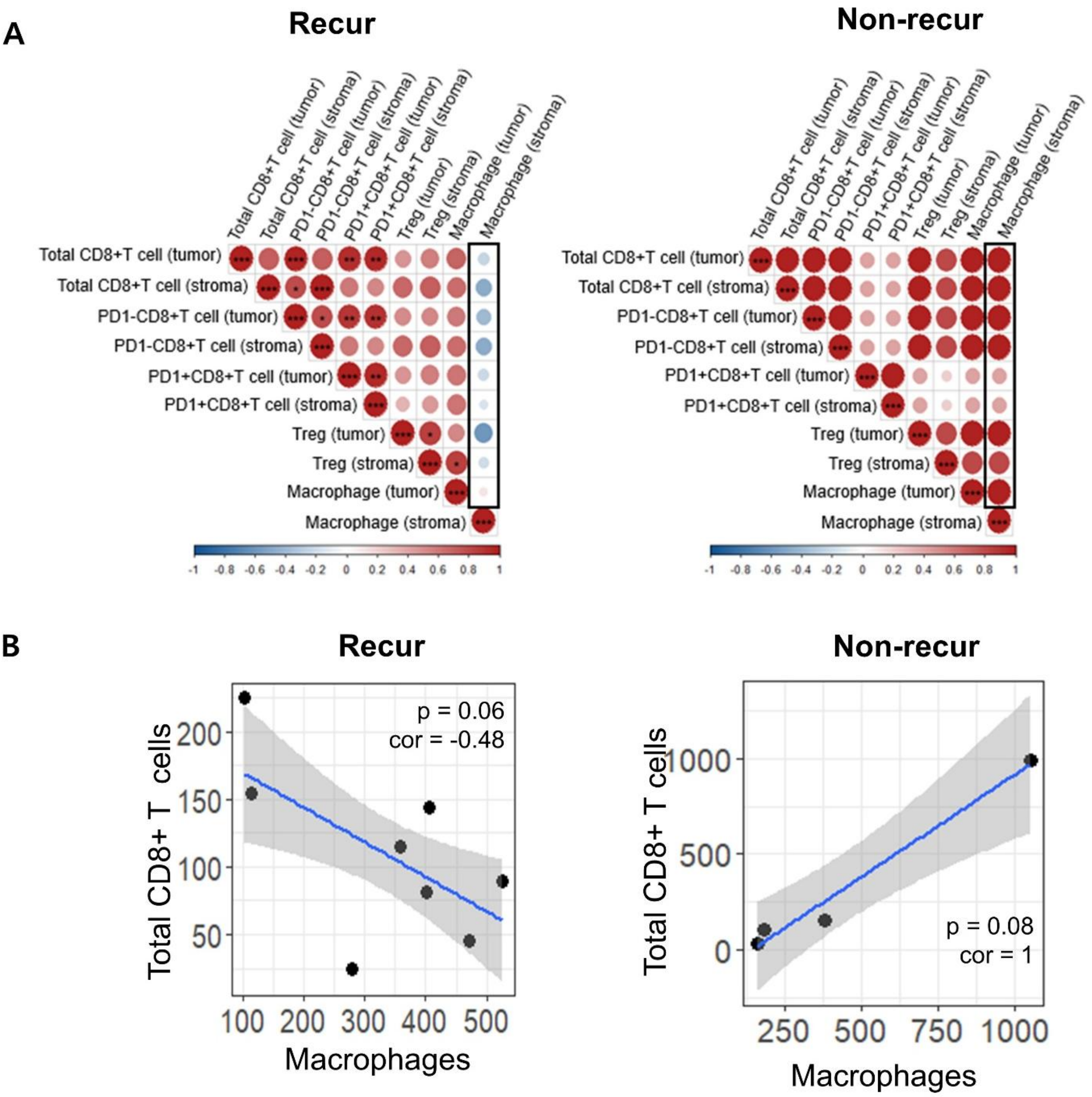

Supplementary Figure 2. Correlation between multispectral immune cell density and recurrence of HGSCs. (A) Plots showing the correlation between multispectral immune cell density and recurrence of HGSC. Statistical significance is denoted by asterisks (* p<0.05, ** p<0.01, *** p<0.001). Colors represent the correlation coefficient: 1 = red; -1 = blue. (B) The correlation between macrophage density and total CD8+ T cell density in the stroma varied according to recurrence status.

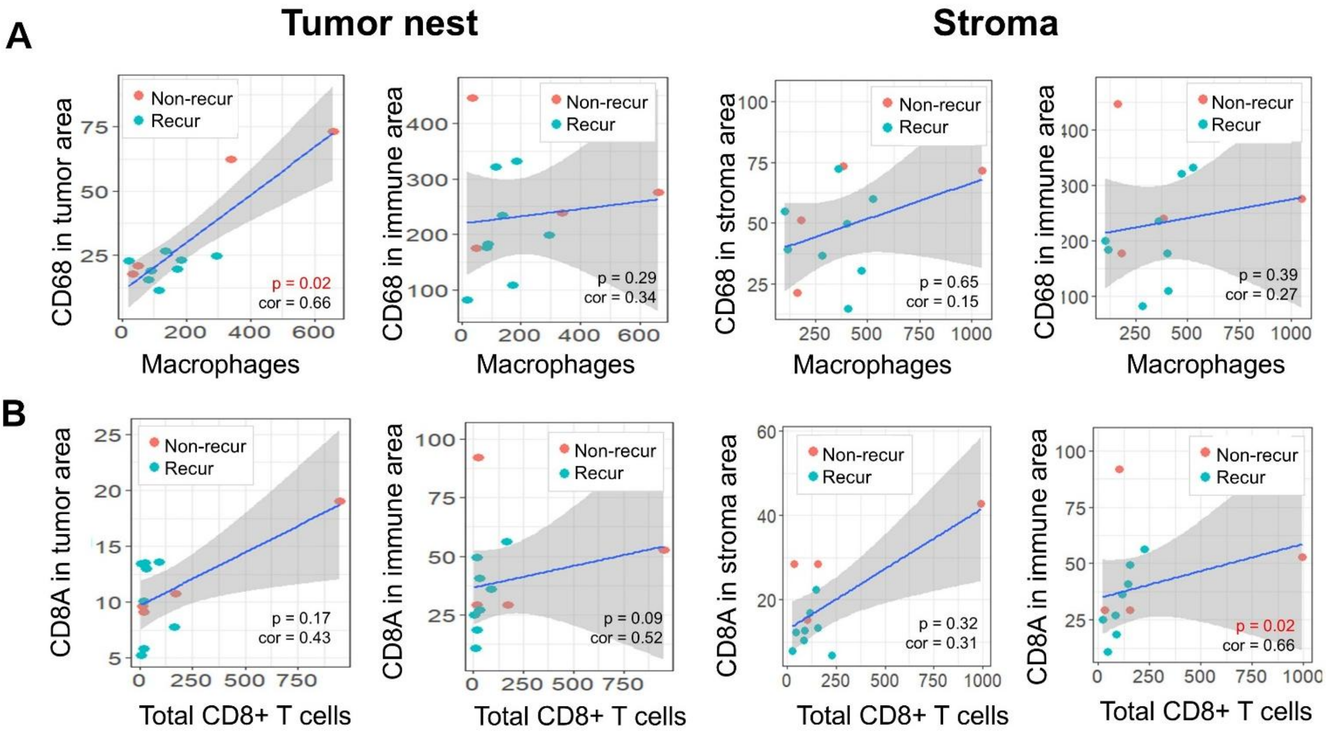

Supplementary Figure3. Expression of immune cell marker genes in GeoMx spatial transcriptome data is similar to the corresponding mIF immune cell densities. (A) Expression of CD68 (a macrophage marker) in the tumor area is similar to the mIF macrophage cell density in the tumor nest (correlation coefficient = 0.66, p = 0.02). The blue line represents the trend of the data, and the gray area denotes the confidence interval for the trend. (B) Expression of CD8A (cytotoxic T cells) in the immune area is similar to the mIF total CD8+ T cell density in the stroma (correlation coefficient = 0.66, p = 0.02).

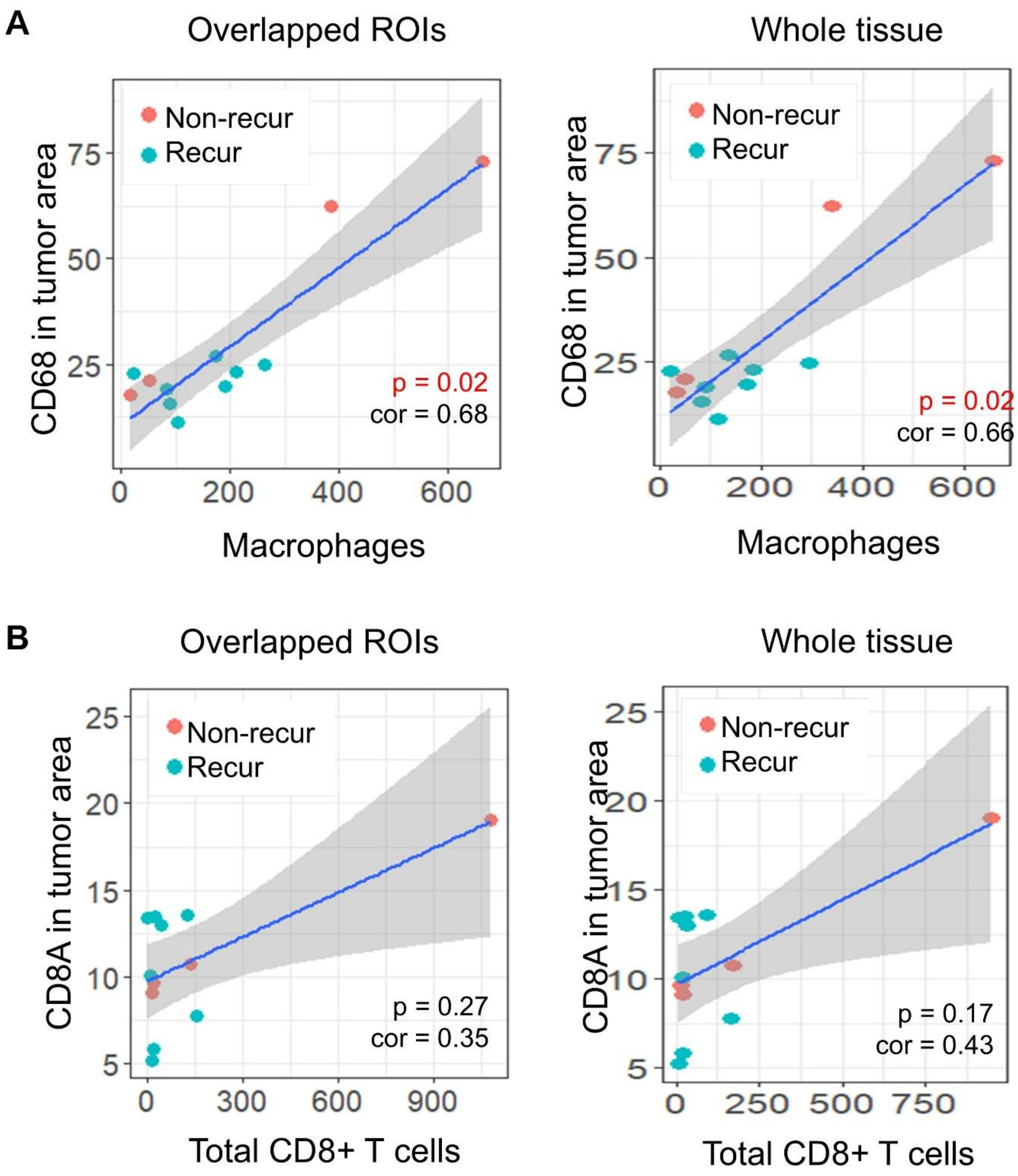

Supplementary Figure 4. Comparison of immune cell densities between overlapping ROIs and the whole tissue in the tumor nest with expression of immune cell marker genes in GeoMx spatial transcriptome data. The correlation between (A) CD68 expression and macrophage immune cell density, and (B) CD8A expression and total CD8+T cell density, in the tumor nest was examined in overlapping ROIs and in the whole tissue; the results were very similar. The blue line represents the trend of the data, and the gray area denotes the confidence interval for the trend
