# Supplementary material for: Identification of Prognostic Biomarkers of Ovarian High-Grade Serous Carcinoma: A Preliminary Study Using Spatial Transcriptome Analysis and Multispectral Imaging
Source: Cells. 2025 May 8;14(10):681. doi: 10.3390/cells14100681 (PMC12110039; doi:10.3390/cells14100681)
Supplement: Supplementary file 1 [file cells-14-00681-s001.zip › HGSC_Geomx_STable.pdf]

Table S1. The proportion (%) of cells in the immune (CD45), tumor (PanCK), and stromal (SMA) areas.

Type	All patients				No-recur group				Recur group				p-value			
	CD45	PanCK	SMA	Total	CD45	PanCK	SMA	Total	CD45	PanCK	SMA	Total	CD45	PanCK	SMA	Total
macrophages	37.59	4.71	14.11	18.80	39.45	9.68	23.80	28.70	36.78	2.54	9.87	14.48	0.82	0.46	0.24	0.015
mast	0.09	1.20	1.23	0.84	0.02	1.62	0.58	0.63	0.13	1.01	1.51	0.93	0.09	0.13	0.44	0.53
B	2.01	13.07	9.68	8.26	1.31	11.83	8.03	7.65	2.33	13.61	10.40	8.52	0.17	1.00	0.65	0.87
plasma	1.13	0.00	4.98	2.03	0.00	0.00	2.60	0.87	1.62	0.00	6.02	2.54	0.08	1.00	0.78	0.55
CD4.T.cells	3.45	22.75	13.23	13.14	2.72	20.68	13.00	15.11	3.76	23.66	13.33	12.28	0.91	0.92	0.97	0.37
CD8.T.cells	29.03	19.36	26.99	25.13	37.27	25.48	24.18	20.06	25.43	16.68	28.21	27.34	0.19	0.22	0.92	0.45
NK	6.32	6.40	8.48	7.07	5.30	5.58	11.18	7.02	6.76	6.76	7.30	7.09	0.36	0.79	0.36	0.97
pDC	0.56	0.72	3.68	1.65	0.36	1.53	1.87	1.24	0.65	0.37	4.48	1.84	0.60	0.11	0.67	0.68
mDCs	1.76	1.67	0.70	1.38	1.44	0.47	1.20	1.25	1.90	2.19	0.48	1.43	0.66	1.00	0.75	0.61
monocytes	9.92	0.37	4.04	4.78	7.93	1.23	2.53	4.47	10.79	0.00	4.71	4.91	0.20	0.03	0.77	0.97
neutrophils	2.01	27.24	11.44	13.56	0.71	21.90	8.91	10.14	2.58	29.57	12.54	15.06	0.04	0.20	0.55	0.41
Treg	6.12	2.51	1.44	3.36	3.51	0.00	2.14	2.86	7.26	3.61	1.14	3.58	0.12	0.08	0.83	0.71

* Statistically significant p-values ($p < 0.05$) were highlighted in red.

Table S2. Differentially expressed genes in each compartment.

**Immune area (CD45)**					
**Gene**	**log2FC**	**FC (fold change)**	**Pvalue**	**Adjusted Pvalue**	**Tumor microenvironment**
SUSD2	5.67	50.76	1.35E-05	0.053	
PIGR	2.63	6.17	0.000212	0.115	
MMP12	2.45	5.46	0.000343	0.126	Matrix remodeling
TFPI2	2.01	4.02	3.51E-05	0.063	
BIRC3	1.90	3.73	0.000271	0.119	
CDKN2A	1.50	2.83	0.000942	0.170	
CTSE	1.45	2.73	2.55E-06	0.016	
ANGPTL5	1.35	2.55	0.000862	0.169	
F13A1	1.34	2.54	0.000862	0.169	

NKG7	1.33	2.51	1.79E-06	0.016	NK cells
SIGLEC10	1.31	2.48	1.38E-06	0.016	
GSTT1	1.28	2.43	3.95E-05	0.063	
BFSP1	1.26	2.40	2.46E-05	0.058	
SLC26A9	1.26	2.39	0.000116	0.093	
CABP7	1.23	2.34	5.24E-05	0.065	
ARL14	1.22	2.32	1.42E-05	0.053	
DUSP13	1.21	2.32	4.22E-05	0.063	
RNF113B	1.20	2.30	0.000252	0.115	
FBXL18	1.19	2.28	0.000235	0.115	
PLA2G7	1.18	2.27	2.52E-05	0.058	
POU3F3	1.17	2.25	0.000118	0.093	
TEX13D	1.16	2.23	0.000377	0.133	
ATOH7	1.15	2.22	0.000212	0.115	
RRH	1.15	2.22	7.77E-05	0.080	
SMPDL3A	1.15	2.21	4.67E-05	0.063	
WNT7B	1.14	2.21	0.000451	0.136	
CYP3A4	1.14	2.20	0.000251	0.115	
ARNTL2	1.14	2.20	7.08E-05	0.078	
KRTAP6-3	1.13	2.19	0.000474	0.136	
CCL18	1.13	2.19	0.000436	0.136	
CYBB	1.13	2.18	1.77E-05	0.055	Immune Suppression by Myeloid Cells
HSPB8	1.12	2.18	8.14E-05	0.080	
TPTE	1.12	2.17	0.000289	0.120	
TNC	1.12	2.17	0.000184	0.115	Matrix
SFTPD	1.11	2.16	0.00024	0.115	
FTMT	1.10	2.14	0.000652	0.156	
WDR47	1.09	2.12	0.000174	0.115	
DRD3	1.08	2.11	0.000402	0.136	
TSGA13	1.08	2.11	0.000323	0.123	

GPR174	1.08	2.11	2.81E-05	0.058	
NUDT10	1.06	2.09	0.000494	0.136	
GNAT3	1.06	2.09	0.000539	0.142	
PRDM15	1.06	2.08	0.000339	0.126	
LCNL1	1.05	2.08	0.000523	0.139	
B3GNTL1	1.05	2.07	0.000774	0.168	
PAK3	1.05	2.07	0.000274	0.119	
DPEP2NB	1.05	2.06	0.00086	0.169	
PBOV1	1.04	2.06	0.000993	0.175	
OTC	1.04	2.06	0.000807	0.169	
ZNF564	1.04	2.05	0.000894	0.169	
FUT4	1.04	2.05	0.000133	0.099	
OR10D3	1.03	2.05	0.000497	0.136	
EN1	1.03	2.04	0.000918	0.169	
ACSF3	1.03	2.04	0.000247	0.115	
RSPO2	1.02	2.03	0.000755	0.168	
AOAH	1.02	2.03	0.000183	0.115	
GM2A	1.02	2.03	0.000102	0.093	
PAQR5	1.02	2.03	0.000625	0.152	
PDHA2	1.02	2.02	0.000697	0.165	
DNAH17	1.01	2.01	0.000766	0.168	
GZMA	1.00	2.00	0.00041	0.136	Effector cells
GGTA1	1.00	2.00	6.63E-05	0.077	
SELENBP1	1.00	2.00	0.000626	0.152	
MYH14	-1.00	0.50	0.000356	0.128	
PBX1	-1.03	0.49	0.000286	0.120	
KLK8	-1.05	0.48	0.000316	0.123	
CCNG1	-1.09	0.47	0.000183	0.115	
EYA2	-1.24	0.42	0.00025	0.115	

Tumor area (PanCK)					
**Gene**	**log2FC**	**FC (fold change)**	**Pvalue**	**Adjusted Pvalue**	**Tumor microenvironment**
TFPI2	3.33	10.08	2.09E-06	0.007	
PIGR	3.16	8.94	4.04E-05	0.018	
RARRES1	2.36	5.13	7.99134E-06	0.010	
ITLN1	2.25	4.74	9.34E-05	0.024	
EZH2	1.76	3.39	8.94E-08	0.002	
NRM	1.76	3.38	4.77E-07	0.004	
TCF19	1.74	3.33	5.17E-06	0.008	
SOX7	1.73	3.31	3.57E-05	0.017	
ID1	1.72	3.30	0.000412865	0.050	
HOXC10	1.70	3.25	9.19E-06	0.011	
DLC1	1.67	3.19	0.000359552	0.047	
H3C8	1.66	3.17	7.95E-05	0.023	
LCP1	1.64	3.13	1.53E-06	0.006	
SYT8	1.63	3.10	2.68E-05	0.015	
CDKN1A	1.62	3.08	0.000921437	0.080	
ZNF596	1.62	3.07	0.000224138	0.038	
H3C7	1.59	3.01	6.48916E-05	0.021	
H2BC9	1.59	3.00	5.16E-05	0.019	
RRM1	1.57	2.97	1.89E-05	0.013	
C12orf75	1.52	2.87	7.64E-06	0.010	
H2AC12	1.50	2.83	1.92E-05	0.013	
ICAM4	1.50	2.83	5.34E-04	0.062	
CDC25B	1.48	2.79	6.76071E-05	0.021	
NCEH1	1.46	2.75	7.30E-05	0.022	
GTF3C5	1.46	2.75	0.000163069	0.033	
CDH5	1.44	2.71	0.000370417	0.047	Angiogenesis
GNG11	1.42	2.68	3.80E-06	0.008	

H2AC17	1.41	2.66	3.05054E-05	0.015	
PLSCR4	1.40	2.65	0.000192397	0.036	
CCDC74A	1.37	2.58	8.75E-05	0.024	
H2BC11	1.36	2.57	2.46E-06	0.007	
TPX2	1.34	2.53	5.37192E-06	0.008	
H2AC13	1.32	2.50	2.14E-05	0.014	
MCM7	1.31	2.48	1.95E-05	0.013	
NSD2	1.30	2.47	1.16E-05	0.011	
MCAM	1.28	2.43	0.000471644	0.055	
NCAPG2	1.27	2.41	6.23E-06	0.009	
ATOH8	1.27	2.41	4.23E-06	0.008	
H2AC11	1.26	2.40	5.00E-05	0.019	
H4C5	1.25	2.37	0.000338956	0.046	
H2BC17	1.22	2.33	3.27683E-05	0.016	
HRAS	1.22	2.33	5.05E-05	0.019	
APBB2	1.20	2.30	2.50E-05	0.015	
SMC4	1.18	2.27	1.06E-04	0.027	
TACC3	1.17	2.26	4.53069E-06	0.008	
GNAI1	1.17	2.25	5.23465E-05	0.019	
CSRP2	1.17	2.25	1.67E-05	0.013	
POLR2L	1.16	2.23	7.23E-04	0.073	
SH3BP2	1.15	2.22	0.000305745	0.042	
MLF1	1.15	2.21	0.000251763	0.040	
INSIG1	1.14	2.21	2.86E-05	0.015	
H2BC10	1.12	2.18	0.000521602	0.061	
SAP25	1.12	2.17	0.000219806	0.038	
MKI67	1.11	2.16	6.88817E-05	0.021	Tumor proliferation rate
ASCL2	1.11	2.16	2.65E-05	0.015	
IGSF10	1.11	2.16	1.70E-04	0.033	
PRSS16	1.11	2.15	6.52E-05	0.021	

HMGB2	1.10	2.14	0.000146379	0.031	
H2BC15	1.10	2.14	9.59401E-05	0.025	
KNTC1	1.10	2.14	1.93818E-05	0.013	
COTL1	1.09	2.13	0.000356189	0.047	
H2BC8	1.09	2.13	3.03E-04	0.042	
ARHGAP11B	1.09	2.13	1.40091E-05	0.012	
CCDC167	1.07	2.11	0.000116501	0.028	
CCNE2	1.07	2.09	1.15564E-05	0.011	
NUP98	1.06	2.09	0.000163656	0.033	
RFC2	1.06	2.08	5.21246E-05	0.019	
E2F1	1.05	2.08	4.49836E-05	0.019	Tumor proliferation rate
H2AC8	1.05	2.07	0.000351469	0.047	
IFT80	1.04	2.06	6.85E-07	0.004	
CDCA7	1.03	2.04	5.77E-05	0.020	
KIF22	1.02	2.03	1.44018E-06	0.006	
TCF7L1	1.02	2.03	0.000146398	0.031	
CPZ	1.02	2.03	9.21E-04	0.080	
CDH13	1.01	2.02	0.000884211	0.079	
SLBP	1.01	2.01	1.29E-04	0.030	
SPECC1	-1.03	0.49	6.41E-04	0.069	
PARP10	-1.04	0.49	0.000618559	0.068	
CA8	-1.09	0.47	0.00037233	0.047	
EXT1	-1.19	0.44	0.000873413	0.079	
ZHX2	-1.19	0.44	0.000810167	0.078	
LRATD2	-1.28	0.41	8.76E-04	0.079	
KCNC3	-1.29	0.41	5.29523E-05	0.019	
SCX	-1.30	0.41	0.000842385	0.079	
GRINA	-1.38	0.38	0.000172831	0.033	
FBXO32	-1.77	0.29	6.14607E-05	0.021	
SST	-1.92	0.26	0.000976702	0.081	

ISG15	-2.25	0.21	0.000733978	0.073	
EYA2	-2.40	0.19	0.000878904	0.079	
IFIT1	-2.48	0.18	0.000815623	0.078	
LYPD1	-2.56	0.17	6.29E-04	0.068	

**Stroma area (SMA)**					
**Gene**	**log2FC**	**FC (fold change)**	**Pvalue**	**Adjusted Pvalue**	**Tumor microenvironment**
MKRN2	2.87	7.33	0.000202	0.188	
TFPI2	2.34	5.07	8.33E-06	0.078	
IGFBP3	2.27	4.81	0.000155	0.161	
RASGRP4	1.98	3.95	0.000389	0.269	
ZNF454	1.68	3.20	4.47E-05	0.093	
C11orf45	1.64	3.12	0.000464	0.271	
ID1	1.58	2.99	3.36E-05	0.090	
A2M	1.51	2.85	1.80E-05	0.090	
COQ10A	1.48	2.79	2.89E-05	0.090	
CDH5	1.38	2.60	4.23E-06	0.078	Angiogenesis
EPO	1.37	2.58	9.52E-05	0.137	
RGS5	1.34	2.53	6.41E-05	0.100	
SLIT2	1.31	2.48	4.33E-05	0.093	
ITLN1	1.27	2.42	0.000521	0.272	
RGCC	1.25	2.38	0.00013	0.149	
TRAC	1.24	2.36	0.00026	0.229	T cells
LRRC15	1.23	2.35	0.000136	0.149	
CD93	1.22	2.34	0.000294	0.229	
HEY1	1.22	2.33	0.000278	0.229	
PLXND1	1.22	2.33	0.000928	0.348	
PCP4	1.22	2.33	0.000406	0.271	
JAG2	1.19	2.28	3.39E-05	0.090	
TNFRSF11B	1.15	2.23	0.000717	0.331	

ARHGDIB	1.15	2.23	2.65E-05	0.090	
EMP1	1.10	2.15	5.05E-05	0.094	
NOTCH4	1.08	2.12	0.000103	0.137	
ACVRL1	1.07	2.10	0.000864	0.343	
ECSCR	1.07	2.10	0.000364	0.262	
H2BC10	1.05	2.07	0.000126	0.149	
SVEP1	1.05	2.06	0.000832	0.343	
ATP13A2	1.05	2.06	0.000525	0.272	
H3C11	1.04	2.06	0.000951	0.348	
TACC2	1.03	2.05	0.000425	0.271	
INSR	1.03	2.04	0.000287	0.229	
F2R	1.03	2.04	0.000487	0.272	
NPDC1	1.01	2.01	5.82E-05	0.099	
RAB5A	-1.00	0.50	0.000944	0.348	
IGF2BP2	-1.04	0.48	0.000839	0.343	
MDK	-1.10	0.47	0.000446	0.271	
ZNF503	-1.22	0.43	0.000846	0.343	
HMGA2	-1.28	0.41	0.000177	0.174	

Table S3. Enriched Geno Ontology terms for up-regulated genes in the tumor (PanCK) area.

Non-recur group

ID	Description	GeneRatio	BgRatio	pvalue	p.adjust
GO:0006334	nucleosome assembly	10/73	126/18800	5.54E-11	7.20E-08
GO:0065004	protein-DNA complex assembly	11/73	203/18800	3.52E-10	1.69E-07
GO:0031497	chromatin assembly	11/73	205/18800	3.91E-10	1.69E-07
GO:0034728	nucleosome organization	10/73	159/18800	5.54E-10	1.80E-07
GO:0071824	protein-DNA complex subunit organization	11/73	237/18800	1.81E-09	4.72E-07
GO:0006338	chromatin remodeling	10/73	266/18800	7.71E-08	1.67E-05
GO:0002385	mucosal immune response	4/73	38/18800	1.40E-05	0.002597

GO:0002251	organ or tissue specific immune response	4/73	41/18800	1.90E-05	0.00309
GO:0000280	nuclear division	9/73	446/18800	5.56E-05	0.008032
GO:0048285	organelle fission	9/73	493/18800	0.000119	0.015525
GO:0140014	mitotic nuclear division	7/73	293/18800	0.000141	0.016634
GO:0002227	innate immune response in mucosa	3/73	27/18800	0.000154	0.016658
GO:1901987	regulation of cell cycle phase transition	8/73	415/18800	0.000202	0.020238
GO:0006335	DNA replication-dependent chromatin assembly	3/73	32/18800	0.000257	0.022286
GO:0034723	DNA replication-dependent chromatin organization	3/73	32/18800	0.000257	0.022286
GO:0044772	mitotic cell cycle phase transition	8/73	440/18800	0.0003	0.024402
GO:0007059	chromosome segregation	7/73	348/18800	0.000401	0.030232
GO:0032200	telomere organization	5/73	162/18800	0.000418	0.030232
GO:0060236	regulation of mitotic spindle organization	3/73	39/18800	0.000464	0.030418
GO:0044843	cell cycle G1/S phase transition	6/73	255/18800	0.000468	0.030418
GO:0000070	mitotic sister chromatid segregation	5/73	171/18800	0.000535	0.033163
GO:0090224	regulation of spindle organization	3/73	43/18800	0.00062	0.036666
GO:0050830	defense response to Gram-positive bacterium	4/73	104/18800	0.000718	0.040592
GO:0045601	regulation of endothelial cell differentiation	3/73	47/18800	0.000806	0.042753
GO:1902806	regulation of cell cycle G1/S phase transition	5/73	188/18800	0.000822	0.042753
GO:0098813	nuclear chromosome segregation	6/73	287/18800	0.000868	0.043437

Recur group

ID	Description	GeneRatio	BgRatio	pvalue	p.adjust
GO:0045071	negative regulation of viral genome replication	3/14	57/18800	9.39E-06	0.004697
GO:0045069	regulation of viral genome replication	3/14	87/18800	3.36E-05	0.006404
GO:0048525	negative regulation of viral process	3/14	91/18800	3.84E-05	0.006404
GO:0007498	mesoderm development	3/14	132/18800	0.000116	0.01121
GO:0019079	viral genome replication	3/14	134/18800	0.000122	0.01121
GO:1903900	regulation of viral life cycle	3/14	139/18800	0.000136	0.01121
GO:0001958	endochondral ossification	2/14	28/18800	0.000193	0.01121
GO:0036075	replacement ossification	2/14	28/18800	0.000193	0.01121
GO:0050792	regulation of viral process	3/14	159/18800	0.000202	0.01121

GO:0042596	fear response	2/14	37/18800	0.000338	0.015486
GO:0007369	gastrulation	3/14	190/18800	0.000341	0.015486
GO:0060350	endochondral bone morphogenesis	2/14	57/18800	0.000803	0.031969
GO:0071357	cellular response to type I interferon	2/14	58/18800	0.000831	0.031969
GO:0030199	collagen fibril organization	2/14	62/18800	0.000949	0.033705
GO:0034340	response to type I interferon	2/14	64/18800	0.001011	0.033705
GO:0001707	mesoderm formation	2/14	73/18800	0.001313	0.038481
GO:0033555	multicellular organismal response to stress	2/14	73/18800	0.001313	0.038481
GO:0048332	mesoderm morphogenesis	2/14	75/18800	0.001385	0.038481
GO:0019058	viral life cycle	3/14	317/18800	0.001506	0.039627
GO:0031397	negative regulation of protein ubiquitination	2/14	84/18800	0.001734	0.043341

* GeneRatio: ratio of input genes annotated within a term

* BgRatio: ratio of all genes annotated within this term

Table S4. Enriched Geno Ontology terms for up-regulated genes in the stroma (SMA) area.

Non-recur group

ID	Description	GeneRatio	BgRatio	pvalue	p.adjust
GO:0045602	negative regulation of endothelial cell differentiation	3/34	12/18800	1.18E-06	0.001
GO:0045601	regulation of endothelial cell differentiation	4/34	47/18800	1.50E-06	0.001
GO:0045446	endothelial cell differentiation	5/34	117/18800	2.06E-06	0.001
GO:0003158	endothelium development	5/34	133/18800	3.88E-06	0.001
GO:0043542	endothelial cell migration	6/34	279/18800	9.60E-06	0.002
GO:0043534	blood vessel endothelial cell migration	5/34	175/18800	1.48E-05	0.003
GO:0044331	cell-cell adhesion mediated by cadherin	3/34	28/18800	1.72E-05	0.003
GO:0001667	ameboidal-type cell migration	7/34	480/18800	2.00E-05	0.003
GO:1901342	regulation of vasculature development	6/34	351/18800	3.51E-05	0.004
GO:0010631	epithelial cell migration	6/34	358/18800	3.92E-05	0.004
GO:0090132	epithelium migration	6/34	361/18800	4.11E-05	0.004
GO:0090130	tissue migration	6/34	366/18800	4.43E-05	0.004

GO:1904019	epithelial cell apoptotic process	4/34	118/18800	5.91E-05	0.005
GO:0003007	heart morphogenesis	5/34	254/18800	8.74E-05	0.007
GO:0030857	negative regulation of epithelial cell differentiation	3/34	50/18800	9.99E-05	0.008
GO:0035907	dorsal aorta development	2/34	10/18800	0.0001	0.010
GO:2000048	negative regulation of cell-cell adhesion mediated by cadherin	2/34	10/18800	0.0001	0.010
GO:2000351	regulation of endothelial cell apoptotic process	3/34	59/18800	0.0002	0.010
GO:0030856	regulation of epithelial cell differentiation	4/34	157/18800	0.0002	0.010
GO:0051056	regulation of small GTPase mediated signal transduction	5/34	299/18800	0.0002	0.010
GO:0035904	aorta development	3/34	62/18800	0.0002	0.010
GO:0046579	positive regulation of Ras protein signal transduction	3/34	62/18800	0.0002	0.010
GO:0007162	negative regulation of cell adhesion	5/34	305/18800	0.0002	0.010
GO:0072577	endothelial cell apoptotic process	3/34	65/18800	0.0002	0.011
GO:0051057	positive regulation of small GTPase mediated signal transduction	3/34	70/18800	0.0003	0.013
GO:0046578	regulation of Ras protein signal transduction	4/34	187/18800	0.0003	0.015
GO:0045765	regulation of angiogenesis	5/34	345/18800	0.0004	0.015
GO:0030336	negative regulation of cell migration	5/34	346/18800	0.0004	0.015
GO:0003184	pulmonary valve morphogenesis	2/34	17/18800	0.0004	0.017
GO:2000146	negative regulation of cell motility	5/34	361/18800	0.0004	0.017
GO:0051271	negative regulation of cellular component movement	5/34	369/18800	0.0005	0.018
GO:1904035	regulation of epithelial cell apoptotic process	3/34	94/18800	0.0006	0.022
GO:0003177	pulmonary valve development	2/34	21/18800	0.0007	0.022
GO:2000047	regulation of cell-cell adhesion mediated by cadherin	2/34	21/18800	0.0007	0.022
GO:0040013	negative regulation of locomotion	5/34	396/18800	0.0007	0.023
GO:0010596	negative regulation of endothelial cell migration	3/34	97/18800	0.0007	0.023
GO:0036303	lymph vessel morphogenesis	2/34	23/18800	0.0008	0.025
GO:2000353	positive regulation of endothelial cell apoptotic process	2/34	24/18800	0.0009	0.026
GO:0008585	female gonad development	3/34	105/18800	0.0009	0.026
GO:0003279	cardiac septum development	3/34	106/18800	0.0009	0.026
GO:0060840	artery development	3/34	106/18800	0.0009	0.026
GO:0032967	positive regulation of collagen biosynthetic process	2/34	25/18800	0.0009	0.026
GO:0046545	development of primary female sexual characteristics	3/34	109/18800	0.0010	0.026

GO:0010714	positive regulation of collagen metabolic process	2/34	26/18800	0.0010	0.026
GO:0010633	negative regulation of epithelial cell migration	3/34	112/18800	0.0011	0.028
GO:0003018	vascular process in circulatory system	4/34	263/18800	0.0012	0.031
GO:0001945	lymph vessel development	2/34	30/18800	0.0013	0.033
GO:0046660	female sex differentiation	3/34	124/18800	0.0014	0.035
GO:0003180	aortic valve morphogenesis	2/34	32/18800	0.0015	0.036
GO:0001569	branching involved in blood vessel morphogenesis	2/34	35/18800	0.0018	0.041
GO:0060284	regulation of cell development	5/34	500/18800	0.0019	0.041
GO:0035909	aorta morphogenesis	2/34	36/18800	0.0019	0.041
GO:0051384	response to glucocorticoid	3/34	139/18800	0.0020	0.041
GO:0003176	aortic valve development	2/34	37/18800	0.0020	0.041
GO:0003203	endocardial cushion morphogenesis	2/34	37/18800	0.0020	0.041
GO:0046326	positive regulation of glucose import	2/34	37/18800	0.0020	0.041
GO:0060485	mesenchyme development	4/34	301/18800	0.0020	0.041
GO:0032147	activation of protein kinase activity	3/34	142/18800	0.0021	0.041
GO:0032965	regulation of collagen biosynthetic process	2/34	38/18800	0.0021	0.041
GO:1904037	positive regulation of epithelial cell apoptotic process	2/34	38/18800	0.0021	0.041
GO:0014912	negative regulation of smooth muscle cell migration	2/34	39/18800	0.0023	0.043
GO:0030513	positive regulation of BMP signaling pathway	2/34	40/18800	0.0024	0.044
GO:0007584	response to nutrient	3/34	150/18800	0.0025	0.044
GO:1902742	apoptotic process involved in development	2/34	41/18800	0.0025	0.044
GO:1905314	semi-lunar valve development	2/34	41/18800	0.0025	0.044
GO:0060412	ventricular septum morphogenesis	2/34	42/18800	0.0026	0.046
GO:0048754	branching morphogenesis of an epithelial tube	3/34	153/18800	0.0026	0.046
GO:0010712	regulation of collagen metabolic process	2/34	43/18800	0.0027	0.046
GO:0060562	epithelial tube morphogenesis	4/34	328/18800	0.0028	0.046
GO:0031960	response to corticosteroid	3/34	157/18800	0.0028	0.046
GO:0010828	positive regulation of glucose transmembrane transport	2/34	44/18800	0.0029	0.046
GO:0045840	positive regulation of mitotic nuclear division	2/34	44/18800	0.0029	0.046
GO:0001837	epithelial to mesenchymal transition	3/34	162/18800	0.0031	0.049
GO:0003197	endocardial cushion development	2/34	47/18800	0.0033	0.050

GO:0032964	collagen biosynthetic process	2/34	47/18800	0.0033	0.050
GO:0003205	cardiac chamber development	3/34	167/18800	0.0034	0.050
GO:0006953	acute-phase response	2/34	48/18800	0.0034	0.050
GO:0035272	exocrine system development	2/34	48/18800	0.0034	0.050
GO:0043114	regulation of vascular permeability	2/34	48/18800	0.0034	0.050
GO:0007265	Ras protein signal transduction	4/34	347/18800	0.0034	0.050

* GeneRatio: ratio of input genes annotated within a term

* BgRatio: ratio of all genes annotated within this term

Table S5. Mean immune cell density according to recurrence status (/㎟).

TME	Recurrence status	Total CD8+T cell	PD1-CD8+T cell	PD1+CD8+T cell	Treg	Macrophage	Treg / Total CD8+T cell	Treg / PD1-CD8+T cell	Treg / PD1+ CD8+T cell	Treg / Macrophage	Macrophage / Total CD8+T cell	Macrophage / PD1-CD8+T cell	Macrophage / PD1+CD8+ T cell
Tumor nest	Non-recur	289.0	134.8	154.3	15.5	270.3	0.03	0.06	0.72	0.03	1.82	2.65	53.54
	Recur	46.6	34.3	12.3	8.4	137.2	0.20	0.23	2.05	0.07	5.16	6.05	46.87
	Recur/Non-recur	0.16	0.25	0.08	0.54	0.51	6.40	4.10	2.84	2.43	2.84	2.28	0.88
	p-value	0.368	0.283	0.683	0.808	0.808	0.016	0.048	0.214	0.283	0.109	0.048	1.00
Stroma	Non-recur	320.7	160.7	160.0	27.9	443.2	0.15	0.20	5.96	0.07	2.52	3.29	86.19
	Recur	110.0	97.8	12.2	32.4	332.5	0.36	0.40	9.51	0.16	5.00	5.54	117.27
	Recur/Non-recur	0.34	0.61	0.08	1.16	0.75	2.37	2.02	1.59	2.39	1.98	1.68	1.36
	p-value	0.570	0.461	0.933	0.570	1.000	0.048	0.109	0.683	0.683	0.368	0.461	1.00

* Non-recur: No-recurrence; Recur: Recurrence; TME: Tumor microenvironment; Treg: regulatory T cells

* Statistically significant p-values (p < 0.05) are highlighted in red.

Table S6. Mean distance of cancer cells to the nearest immune cells (μm).

Recurrence status	Total CD8+ T cell	PD1-CD8+ T cell	PD1+CD8+T cell	Treg	Macrophage	Treg / Total CD8+T cell	Treg / PD1-CD8+ T cell	Treg / PD1+CD8+ T cell	Treg / Macrophage	PD1+CD8+ T cell / PD1-CD8+ T cell	Macrophage / Total CD8+T cell	Macrophage / PD1-CD8+T cell	Macrophage / PD1+ CD8+T cell
**Non-recur**	105.2	114.4	257.8	248.9	57.2	3.2	2.6	1.7	4.7	2.5	0.70	0.57	0.40
**Recur**	147.2	157.4	275.5	215.7	57.7	1.6	1.5	0.9	4.2	1.8	0.42	0.38	0.23
**Recur/Non-recur**	1.40	1.38	1.07	0.87	1.01	0.51	0.55	0.52	0.89	0.72	0.61	0.66	0.58
**p-value**	0.461	0.570	0.933	0.683	0.683	0.048	0.048	0.461	0.283	1.000	0.214	0.154	0.683

* Non-recur: No-recurrence;Recur: Recurrence; TME: Tumor microenvironment; Treg: regulatory T cells.

* Statistically significant p-values ($p < 0.05$) are highlighted in red.

Table S7. Mean distance of macrophages to the nearest immune cell (μm).

TME compartment	Recurrence status	Total CD8+T cell	PD1-CD8+T cell	PD1+CD8+T cell	Treg	Treg / Total CD8+T cell	Treg / PD1-CD8+T cell	Treg / PD1+CD8+T cell
**Tumor nest**	**Non-recur**	137.1	146.4	254.7	317.8	3.01	2.50	1.87
	**Recur**	194.9	210.3	271.1	279.1	1.59	1.42	1.05
	**Recur/Non-recur**	1.42	1.44	1.06	0.88	0.53	0.57	0.56
	**p-value**	0.368	0.368	0.808	0.808	0.048	0.028	0.570
**Stroma**	**Non-recur**	128.5	142.4	247.1	250.6	2.51	2.01	1.48
	**Recur**	132.0	140.5	259.0	190.8	1.50	1.39	0.77
	**Recur/Non-recur**	1.03	0.99	1.05	0.76	0.60	0.69	0.52
	**p-value**	0.933	0.933	0.683	0.154	0.368	0.214	0.283

* No-Recur: No-recurrence; Recur: Recurrence; TME: Tumor microenvironment; Treg: regulatory T cells.

* Statistically significant p-values ($p < 0.05$) are highlighted in red.
